# Improving Industrially Relevant Phenotypic Traits by Engineering Chromosome Copy Number in *Saccharomyces pastorianus*

**DOI:** 10.3389/fgene.2020.00518

**Published:** 2020-06-03

**Authors:** Arthur R. Gorter de Vries, Ewout Knibbe, Roderick van Roosmalen, Marcel van den Broek, Pilar de la Torre Cortés, Stephanie F. O’Herne, Pascal A. Vijverberg, Anissa el Masoudi, Nick Brouwers, Jack T. Pronk, Jean-Marc G. Daran

**Affiliations:** Department of Biotechnology, Delft University of Technology, Delft, Netherlands

**Keywords:** *Saccharomyces pastorianus*, chromosome missegregation, chromosome copy number stability, strain engineering, lager beer brewing

## Abstract

The lager-brewing yeast *Saccharomyces pastorianus* is a hybrid between *S. cerevisiae* and *S. eubayanus* with an exceptional degree of aneuploidy. While chromosome copy number variation (CCNV) is present in many industrial *Saccharomyces* strains and has been linked to various industrially-relevant traits, its impact on the brewing performance of *S. pastorianus* remains elusive. Here we attempt to delete single copies of chromosomes which are relevant for the production of off-flavor compound diacetyl by centromere silencing. However, the engineered strains display CNV of multiple non-targeted chromosomes. We attribute this unintended CCNV to inherent instability and to a mutagenic effect of electroporation and of centromere-silencing. Regardless, the resulting strains displayed large phenotypic diversity. By growing centromere-silenced cells in repeated sequential batches in medium containing 10% ethanol, mutants with increased ethanol tolerance were obtained. By using CCNV mutagenesis by exposure to the mitotic inhibitor MBC, selection in the same set-up yielded even more tolerant mutants that would not classify as genetically modified organisms. These results show that CCNV of alloaneuploid *S. pastorianus* genomes is highly unstable, and that CCNV mutagenesis can generate broad diversity. Coupled to effective selection or screening, CCNV mutagenesis presents a potent tool for strain improvement.

## Introduction

The lager brewing yeast *Saccharomyces pastorianus* is an interspecific hybrid of *Saccharomyces cerevisiae* and the cold-tolerant *Saccharomyces eubayanus* (Libkind et al., 2011; Salazar et al., 2019). Chromosomes from both parental species are present in the genome of *S. pastorianus* in varying number of copies, making the genome alloploid and aneuploid (Dunn and Sherlock, 2008). Quantitative measurement based on a combination of sequencing data and flow cytometry based DNA quantification estimated the chromosome copy number ranging from 49 to 79 chromosomes in *S. pastorianus*, a quantification that contributed to distinguish two groups based on copy number of chromosomes from each parental species. Although all *S. pastorianus* strains have an approximately diploid *S. eubayanus* subgenome, Group 1 strains (Saaz) have a, generally incomplete, haploid *S. cerevisiae* subgenome, while Group 2 strains (Frohberg) have a diploid or higher *S. cerevisiae* subgenome (Nakao et al., 2009; van den Broek et al., 2015; Okuno et al., 2016). Reflecting these differences in genome composition, Group 1 strains are more cold-tolerant whereas Group 2 strains exhibit more efficient maltotriose consumption, traits associated with *S. eubayanus* and *S. cerevisiae*, respectively (Gibson et al., 2013; Brouwers et al., 2019a, b). Chromosome recombinations at the *ZUO1*, *MAT*, *HSP82*, and *XRN1*/*KEM1* loci which were found in all *S. pastorianus* isolates suggest that they evolved from a single hybrid ancestor, and that the extensive CCNV of *S. pastorianus* strains emerged during its domestication (Hewitt et al., 2014; Walther et al., 2014; van den Broek et al., 2015; Okuno et al., 2016; Gorter de Vries et al., 2019b). Moreover, there are large copy number differences between the genomes of *S. pastorianus* strains, even among supposedly clonal isolates, suggesting high genomic plasticity and chromosome copy number instability (Bolat et al., 2008; van den Broek et al., 2015).

Chromosomal copy number variation (CCNV) in yeast is generally caused by chromosome missegregation during mitosis. Normally, chromosome segregation during anaphase is ensured by attachment of the microtubule spindle to the kinetochore. Despite numerous control mechanisms (checkpoints) for correct kinetochore attachment, cells can proceed to anaphase while chromosomes are incorrectly attached, leading to gain and loss of a chromosome copy in the resulting daughter cells (Musacchio and Salmon, 2007). The rate at which chromosome missegregation occurs is increased in polyploid and aneuploid genomes, resulting in increased chromosomal copy number instability (Sheltzer et al., 2011; Storchova, 2014). Changes in chromosome copy number are generally reflected by altered expression levels of genes on the affected chromosome and the correspondingly altered protein levels can cause significant phenotypic effects (Pavelka et al., 2010; Dephoure et al., 2014). In accordance with the greater phenotypic impact of copy number than SNPs in *Saccharomyces* strains (Peter et al., 2018), CCNV can be beneficial under specific selective conditions due to the effect of single or multiple affected genes (Gorter de Vries et al., 2017). Indeed, spontaneous chromosome gain and loss are common in *Saccharomyces* yeast strains derived from mutation accumulation experiments, laboratory evolution studies and industrial settings (Zhu et al., 2014; Hose et al., 2015; Gorter de Vries et al., 2017, 2019c). In an evolutionary context aneuploidy and CCNV appear to have a role in creating large phenotypic diversity in a population and thereby allowing large phenotypic leaps (Chen et al., 2012). However, when introducing CCNV in euploid strains, aneuploidy causes deleterious effects such as increased genome instability, low sporulation efficiency, reduced growth rate, increased nutrient uptake rates, and reduced replicative life span, which are jointly referred to as the aneuploidy-associated stress response (AASR). AASR was attributed to an imbalance of gene expression, specifically of protein complexes, and to overloading of the protein degradation pathways (Torres et al., 2011; Santaguida and Amon, 2015b). Due to the combination of beneficial and detrimental effects of CCNV, aneuploidy can be a transient adaptation to stress which is subsequently replaced by mutations with less side effects after continued evolution (Yona et al., 2012). However, in many wild and industrial yeasts, no direct detrimental effects from aneuploidy are reported and the typical AASR is not observed (Hose et al., 2015), indicating that cells can adapt to minimize the negative impact of aneuploidy while still benefiting from the phenotypic diversity generated by CCNV.

The CCNV in lager brewing yeast *S. pastorianus* affects more chromosomes than the few chromosomes typically observed after laboratory evolution or in natural isolates (Brickwedde et al., 2017). As the current CCNV in *S. pastorianus* strains emerged under the selective pressure of the lager brewing environment and is apparently stably maintained, it likely contributes to its performance in this environment. Indeed, CCNV correlates with industrially-relevant traits such as flocculation and diacetyl production in otherwise nearly isogenic *S. pastorianus* strains (van den Broek et al., 2015). However, to determine if CCNV is causal for flocculation and diacetyl production, a method to engineer CCNV in *S. pastorianus* is required to generate a library of isogenic strains which differ only in CCNV. In *S. cerevisiae* strains, targeted gain or loss of a single chromosome copy can be achieved by introducing an inducible promotor and a counter-selectable marker gene immediately adjacently to a centromere through homologous recombination (Hill and Bloom, 1987). Strong promoter expression disrupts centromere function, increasing the frequency of missegregation of sister chromatids and leading to daughter cells which either a lost or a duplicated chromosome (Panzeri et al., 1984). Cells that lost or duplicated the chromosome can be selected based on selection or counter-selection of an inserted marker. Such conditional centromeres have been used to construct diploid *S. cerevisiae* strains hemizygous or disomic for various chromosomes (Reid et al., 2008; Anders et al., 2009).

In this study, conditional centromeres were introduced and induced in *S. pastorianus* Group 2 strain CBS 1483 in order to create isogenic *S. pastorianus* strains differing only by their CCNV. Brewing relevant phenotypes of the resulting strains were characterized to investigate the effect of CCNV in the complex aneuploid genome of *S. pastorianus*. In addition, CCNV stability of CBS 1483 during growth and during genetic manipulation was evaluated. Finally the potential of inducing CCNV using centromere-silencing or chemical mutagenesis to generate phenotypic diversity was investigated by selecting for strains with increased ethanol tolerance.

## Materials and Methods

### Yeast Strains and Media

The *Saccharomyces* strains used in this study are indicated in Table 1. *S. pastorianus* strain CBS 1483 was obtained from the Westerdijk Fungal Biodiversity Institute.^1^ Yeast strains and *E. coli* strains containing plasmids were stored at −80°C in 30% glycerol (vol/vol). For preparation of stock cultures and inocula of bioreactors, yeast strains were routinely propagated in shake flasks containing 100 mL YPD (10 g/L yeast extract, 20 g/L yeast peptone and 20 g/L glucose) at 30°C and 200 RPM in an Brunswick Innova43/43R shaker (Eppendorf Nederland B.V., Nijmegen, Netherlands). For cultivation on solid media, YPD medium was supplemented with 20 g/L Bacto agar (Becton Dickinson, Breda, Netherlands) and incubation was done at 30°C. Synthetic medium (SM), containing 3 g/L KH_2_PO_4_, 0.5 g/L MgSO_4_.7H_2_O, 5 g/L (NH_4_)_2_SO_4_, 1 mL/L of a trace element solution and 1 mL/L of a vitamin solution, was prepared as previously described (Bruinenberg et al., 1983; Verduyn et al., 1992). For growth in the presence of ethanol, absolute ethanol (Sigma Aldrich, St. Louis, MO, United States) was added in varying concentration to YPD or SMD (20 with g/L glucose) with a correspondingly decreased volume of water to avoid dilution of medium components. Selection for the *amdS* marker was performed on SM-AC: SM with 0.6 g/L acetamide and 6.6 g/L K_2_SO_4_ instead of (NH_4_)_2_SO_4_ as nitrogen source (Solis-Escalante et al., 2013). Induction of the *GAL1* promoter (*GAL1*_p_) was performed on YPGal medium (10 g/L yeast extract, 20 g/L yeast peptone and 20 g/L galactose) or on SMGal-AC medium: SM-AC medium with 20 g/L galactose instead of glucose. For counter selection of the *amdS* marker, strains were first grown on YPD and then on SMD-FAC: SMD supplemented with 2.3 g/L fluoroacetamide (Solis-Escalante et al., 2013). Industrial wort was provided by HEINEKEN Supply Chain B.V. (Zoeterwoude, Netherlands), and contained 14.4 g/L glucose, 2.3 g/L fructose, 85.9 g/L maltose, 26.8 g/L maltotriose, and 269 mg/L free amino nitrogen (Brickwedde et al., 2017). The wort was supplemented with 1.5 mg/L Zn^2+^ by addition of ZnSO_4_.7H_2_O, then autoclaved for 30 min at 121°C and, prior to use, filtered through Nalgene 0.2 μm SFCA bottle-top filters (ThermoFisher Scientific, Waltham, MA, United States). For experiments performed with diluted wort, two volumes of sterile demineralized water were added per volume of wort. To prevent excessive foaming during the aeration phase of the bioreactor experiments, (un)diluted wort was supplemented with 0.2 mL/L of sterile Pluronic PE 6100 antifoam (Sigma-Aldrich).

**TABLE 1:** *Saccharomyces pastorianus* strains used in this study.

Strain Name	Host strain	Description or intended mutation	Sequenced genotype	References
CBS 1483	–	Group 2 strain	wildtype	(van den Broek et al., 2015; Brickwedde et al., 2017)
IMI350	CBS 1483	*SeCEN6::amdS-GAL1p*	Not sequenced	This study
IMS349	CBS 1483	SeCHRVI^–1^	SeCHRVI^–^^1^	This study
IMI351	IMI350	SeCHRVI^–1^	*Sc*CHRV^–1^ *Sc*CHRX-*Se*CHRX^+1^ *Se*CHRVI^–^^1^ *Se*CHRX*-Sc*CHRX^–1^	This study
IMI352	CBS 1483	*ScCEN9*::*amdS-GAL1*p	Not sequenced	This study
IMI353	IMI352	*Sc*CHRIX^–1^	*Sc*CHRXV-XI^–1^	This study
IMI359	CBS 1483	*Sc*CEN10:: *amdS-GAL1*p	Not sequenced	This study
IMI360	CBS 1483	*Sc*CEN12:: *amdS-GAL1*p	Not sequenced	This study
IMI361	CBS 1483	*Sc*CEN14:: *amdS-GAL1*p	*Sc*CEN14:: *amdS-GAL1*p *Sc*CHRII^–1^ *Sc*CHRVIII^–1^ SeCHRVII-ScCHRVII^–1^	This study
IMI363	CBS 1483	*Se*CEN3:: *amdS-GAL1*p	Not sequenced	This study
IMI366	CBS 1483	*Se*CEN8:: *amdS-GAL1*p	Not sequenced	This study
IMI367	CBS 1483	*Se*CEN10:: *amdS-GAL1*p	Not sequenced	This study
IMI368	CBS 1483	*Se*CEN12:: *amdS-GAL1*p	Not sequenced	This study
IMI369	CBS 1483	*Se*CEN14:: *amdS-GAL1*p	Not sequenced	This study
IMI373	IMI359	*Sc*CHRX-*Se*CHRX^–^^1^	*Sc*CHRX-*Se*CHRX^–^^1^ *Se*CHRIX	This study
IMI374	IMI360	*Sc*CHRXII^–^^1^	*Sc*CHRII *Sc*CHRVIII^–1^ *Sc*CHRXII^–^^1^ *Se*CHRIX^–1^	This study
IMI375	IMI361	*Sc*CHRXIV^–^^1^	*Sc*CHRII^–1^ *Sc*CHRV^–1^ *Sc*CHRVIII^–3^ *Sc*CHRXIII^–1^ *Sc*CHRXIV^–^^1^ *Se*CHRI^–1^ *Se*CHRIII-*Sc*CHRIII^–1^ SeCHRVII-ScCHRVII^–1^ SeCHRVIII-XV^+1^	This study
IMI377	IMI363	*Se*CHRIII^–1^	*Sc*CHRI^1^ *Sc*CHRVIII^–1^ *Se*CHRIX^–1^	This study
IMI380	IMI366	*Se*CHRVIII^–1^	*Sc*CHRV^–1^ *Sc*CHRVIII^–2^ SeCHRIX^+2^	This study
IMI381	IMI367	*Se*CHRX^–^^1^	*Sc*CHRIV^+1^ *Sc*CHRVIII^–1^ *Sc*CHRIX^+1^ *Sc*CHRXI^–1^ *Sc*CHRXII^–1^ *Se*CHRII-IV^–1(D)^ *Se*CHRX-*Sc*CHRX^–^^1^ *Se*CHRXI^+1^	This study
IMI382	IMI368	*Se*CHRXII^–^^1^	*Sc*CHRIV^–1^ *Sc*CHRV^–1^ *Sc*CHRVIII^–1^ *Sc*CHRX-*Se*CHRX^–1^ *Se*CHRVI^–1^ *Se*CHRXII^–^^1^ *Se*CHRXV-VIII^–1(D)^	This study
IMI383	IMI369	*Se*CHRXIV^–1^	*Sc*CHRX-*Se*CHRX^–1^ *Se*CHRIX^–1^	This study
IMS0687	CBS 1483	MBC mutagenesis reactor MBC1	*Sc*CHRI^–1^ *Sc*CHRV^–1^ *Sc*CHRXIII^–1^ *Se*CHRIX^+1^ *Se*CHRX-ScCHRX^–1^ *Se*CHRXIV^–1^ *Se*CRXV-VIII^–1^	This study
IMS0688	CBS 1483	MBC mutagenesis reactor MBC1	Not sequenced	This study
IMS0689	CBS 1483	MBC mutagenesis reactor MBC1	Not sequenced	This study
IMS0690	CBS 1483	MBC mutagenesis reactor MBC1	Not sequenced	This study
IMS0691	CBS 1483	MBC mutagenesis reactor MBC1	Not sequenced	This study
IMS0692	CBS 1483	MBC mutagenesis reactor MBC1	Not sequenced	This study
IMS0693	CBS 1483	MBC mutagenesis reactor MBC1	Not sequenced	This study
IMS0694	CBS 1483	MBC mutagenesis reactor MBC1	Not sequenced	This study
IMS0695	CBS 1483	MBC mutagenesis reactor MBC1	Not sequenced	This study
IMS0696	CBS 1483	MBC mutagenesis reactor MBC1	Not sequenced	This study
IMS0697	CBS 1483	MBC mutagenesis reactor MBC1	Not sequenced	This study
IMS0698	CBS 1483	MBC mutagenesis reactor MBC1	Not sequenced	This study
IMS0699	CBS 1483	MBC mutagenesis reactor MBC1	Not sequenced	This study
IMS0700	CBS 1483	MBC mutagenesis reactor MBC1	Not sequenced	This study
IMS0701	CBS 1483	MBC mutagenesis reactor MBC1	Not sequenced	This study
IMS0702	CBS 1483	MBC mutagenesis reactor MBC1	Not sequenced	This study
IMS0703	CBS 1483	MBC mutagenesis reactor MBC1	*Sc*CHRI^–1^ *Sc*CHRV^–1^ *Sc*CHRVIII^–1^ *Sc*CHRXIII^–1^ *Se*CHRIX^–1^ *Se*CHRXIV^–1^ *Se*CRXV-VIII^–1^	This study
IMS0704	CBS 1483	MBC mutagenesis reactor MBC1	Not sequenced	This study
IMS0705	CBS 1483	Electroporated	*Sc*CHRVIII^–1^ *Se*CHRVI^+1^ *Se*CHRX-ScCHRX^–1^	This study
IMS0706	CBS 1483	Electroporated	*Sc*CHRI^–1^ *Sc*CHRVIII^–1^ *Se*CHRIX^–1^	This study
IMS0707	CBS 1483	Electroporated	Not sequenced	This study
IMS0708	CBS 1483	Electroporated	*Sc*CHRI^–1^ *Se*CHRX-*Sc*CHRX^–1^ *Se*CHRXVI^–1^	This study
IMS0709	CBS 1483	Electroporated	*Sc*CHRI^–1^ *Sc*CHRVIII^–1^ SeCHRIX^–1^	This study
IMS0710	CBS 1483	Restreaked untransformed	ploidy unchanged	This study
IMS0711	CBS 1483	Restreaked untransformed	*Se*CHRVII-*Sc*CHRVII^+1^	This study
IMS0712	CBS 1483	Restreaked untransformed	*Se*CHRX-ScCHRX^–1^	This study
IMS0713	CBS 1483	Restreaked untransformed	Not sequenced	This study
IMS0714	CBS 1483	Restreaked untransformed	*Sc*CHRIX^–1^	This study
IMS0715	CBS 1483	MBC mutagenesis reactor MBC2	Not sequenced	This study
IMS0716	CBS 1483	MBC mutagenesis reactor MBC2	*Sc*CHRI^–1^ *Sc*CHRV^–1^ *Sc*CHRVII^+1^ ScCHRVIII^–2^ *Sc*CHRXV-XI^–1^ *Se*CHRVII-ScCHRVII^–1^ *Se*CHRXIV^–1^ *Se*CRXV-VIII^–1^	This study
IMS0717	CBS 1483	MBC mutagenesis reactor MBC2	Not sequenced	This study
IMS0718	CBS 1483	MBC mutagenesis reactor MBC2	Not sequenced	This study
IMS0719	CBS 1483	MBC mutagenesis reactor MBC2	Not sequenced	This study
IMS0720	CBS 1483	MBC mutagenesis reactor MBC2	Not sequenced	This study
IMX1875	IMI361	Galactose induction mutagenesis reactor GAL1	Not sequenced	This study
IMX1876	IMI361	Galactose induction mutagenesis reactor GAL1	Not sequenced	This study
IMX1877	IMI361	Galactose induction mutagenesis reactor GAL1	Not sequenced	This study
IMX1878	IMI361	Galactose induction mutagenesis reactor GAL1	Not sequenced	This study
IMX1879	IMI361	Galactose induction mutagenesis reactor GAL1	Not sequenced	This study
IMX1880	IMI361	Galactose induction mutagenesis reactor GAL1	Not sequenced	This study
IMX1881	IMI361	Galactose induction mutagenesis reactor GAL1	Not sequenced	This study
IMX1882	IMI361	Galactose induction mutagenesis reactor GAL1	Not sequenced	This study
IMX1883	IMI361	Galactose induction mutagenesis reactor GAL1	Not sequenced	This study
IMX1884	IMI361	Galactose induction mutagenesis reactor GAL1	Not sequenced	This study
IMX1885	IMI361	Galactose induction mutagenesis reactor GAL1	Not sequenced	This study
IMX1886	IMI361	Galactose induction mutagenesis reactor GAL1	Not sequenced	This study
IMX1887	IMI361	Galactose induction mutagenesis reactor GAL1	Not sequenced	This study
IMX1888	IMI361	Galactose induction mutagenesis reactor GAL1	Not sequenced	This study
IMX1889	IMI361	Galactose induction mutagenesis reactor GAL1	Not sequenced	This study
IMX1890	IMI361	Galactose induction mutagenesis reactor GAL2	Not sequenced	This study
IMX1891	IMI361	Galactose induction mutagenesis reactor GAL2	Not sequenced	This study
IMX1892	IMI361	Galactose induction mutagenesis reactor GAL2	Not sequenced	This study
IMX1893	IMI361	Galactose induction mutagenesis reactor GAL2	Not sequenced	This study

*For each strain, the strain from which it was derived is indicated, and the genotype is indicated for strains which were sequenced.*

### Analytical Methods and Statistics

Optical density at 660 nm was measured with a Libra S11 spectophotometer (Biochrom, Cambridge, United Kingdom). HPLC analysis of sugar and metabolite concentrations was performed with an Agilent Infinity 1260 chromatography system (Agilent Technologies, Santa Clara, CA, United States) with an Aminex HPX-87 column (Bio-Rad, Lunteren, Netherlands) at 65°C, eluted with 5 mM H_2_SO_4_ (Diderich et al., 2018). Vicinal diketone concentrations (diacetyl and 2,3 pentanedione) were measured using static headspace gas chromatography in a 7890A Agilent GC (Agilent) with an electron capture detector on a CP-Sil 8 CB capillary column, prior to injection 450 μl of supernatant was mixed with 50 μl of 1 mg/L 2,3 hexanedione which acts as an internal standard, and samples were pre-heated for 30 min to 65°C (Brickwedde et al., 2017). Injection was performed with a CTC Combi Pal headspace autoinjector (CTC analytics AG, Zwingen, Switzerland). Significance of data was assessed by an unpaired two-tailed Student’s *t*-test with a 95% confidence interval.

### Genomic DNA Extraction, Whole Genome Sequencing and Analysis

Yeast cultures were inoculated from frozen stocks into 500 mL shake flasks containing 100 mL liquid YPD medium and incubated at 12°C on an orbital shaker set at 200 rpm until the strains reached stationary phase with an OD_660_ between 12 and 20. Genomic DNA was isolated using the Qiagen 100/G kit (Qiagen, Hilden, Germany) according to the manufacturer’s instructions and quantified using a Qubit^®^ Fluorometer 2.0 (ThermoFisher Scientific, Waltham, MA, United States). Genomic DNA of CBS 1483, IMI349, IMI351, IMI353, IMS0687, IMS703, IMS705, IMS706, IMS708-IMS714, IMS716 was sequenced at Novogene Bioinformatics Technology Co., Ltd (Yuen Long, Hong Kong) on a HiSeq2500 sequencer (Illumina, San Diego, CA, United States) with 150 bp paired-end reads using PCR-free library preparation (Brickwedde et al., 2018). Genomic DNA of IMI363, IMI373, IMI374, IMI375, IMI377, IMI370, IMI381, IMI382, IMI383 was sequenced in house on a MiSeq sequencer (Illumina) with 300 bp paired-end reads using PCR-free library preparation.

More than 3 Gb of data per strain representing a minimum of 50-fold coverage of the aneuploid genome of the *S. pastorianus* were generated. Sequence reads of each strain were mapped onto *S. pastorianus* CBS 1483 sequence [genome PRJNA522669, (Salazar et al., 2019)] using the Burrows–Wheeler Alignment tool (BWA) and further processed using SAMtools (Li and Durbin, 2009, 2010). Single-nucleotide variations and indels were determined using Pilon (Walker et al., 2014) based on the BWA.bam output file. The Pilon results file.vcf was visualized using the Integrative Genomics Viewer IGV4. All Illumina sequencing data are available at NCBI^2^ under the bioproject accession number PRJNA522669 and PRJNA612191. Prediction of chromosome copy number was performed using Magnolya, as previously described in Nijkamp et al. (2012).

The variant calling files (vcf) for strains IMS0687, IMS0703, and IMS0716 are available at the 4TU Centre for data research^3^ under the Digital Object Identifier doi: 10.4121/uuid:e5bc2cfe-d726-44a1-bc0a-d3a06653694b.

### Plasmids Construction

**FIGURE 1:**
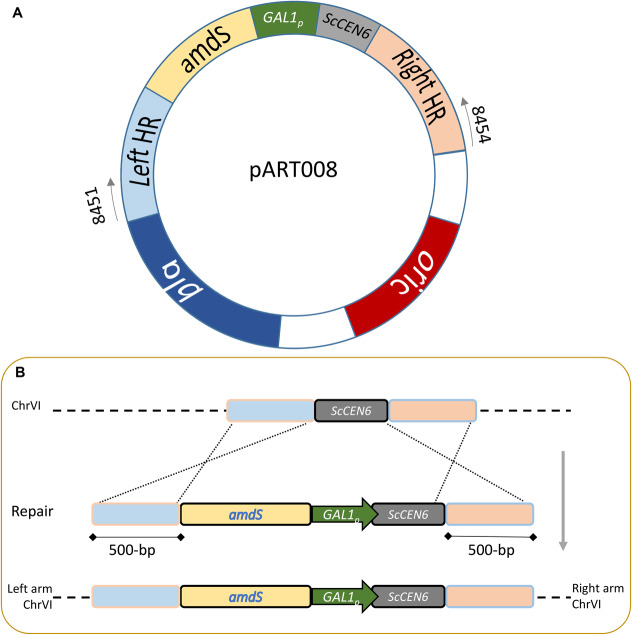
Centromere silencing strain construction. **(A)** The plasmid pART series illustrated by pART008 is designed to introduce the amdS selection marker and the constitutive strong promoter *GAL1* (*GAL1*_p_) upstream a centromere (*Sc*CEN6 in the case of pART008). In addition pART plasmids contain an E. coli replication origin from pBR322 and the bla gene conferring resistance to β-lactam antibiotics. **(B)** Integration of the centromere silencing construct at the *Sc*CEN6 locus in *S. pastorianus* CBS 1483. Schematic representation of the integration of *amdS* selection marker and the *GAL1* promoter containing linear fragment amplified with primers 8451 and 8454. The integration is directed by homology regions of ∼500 bp to complete the double cross over integration.

All plasmids were propagated in *E. coli* DH5α (Table 2). Primers were ordered at Sigma Aldrich (Supplementary Table S1). pART001 was constructed using the NEBuilder^®^ HiFi DNA Assembly method with the backbone of pUG-amdSYM, amplified using primers 8624 & 8556, and the *GAL1* promoter of pAG426GAL (pAG426GAL Addgene plasmid # 14155; http://n2t.net/addgene:14155) (Alberti et al., 2007) that was amplified using primers 8623 & 8436. Genomic DNA of CBS 1483 was extracted with the YeaStarTM Genomic DNA kit (Zymo Research, Irvine, CA, United States) according to Protocol I supplied by the manufacturer. For each targeted centromere, 1000 bp just downstream of the centromere and 1000 bp including the centromere were PCR amplified from genomic DNA of CBS 1483, using primer overhangs for plasmid construction using the NEBuilder^®^ HiFi DNA Assembly method. Homologous sequences were amplified using primers pairs 9857 & 9858 and 9859 & 9860 for *Sc*CEN8, primers pairs 8443 & 8444 and 8557 & 8510 for *Sc*CEN9, primers pairs 10088 & 10089 and 9863 & 9864 for *Sc*CEN10, primers pairs 9865 & 9866 and 9867 & 9868 for *Sc*CEN12, primers pairs 9869 & 9870 and 9871 & 9872 for *Sc*CEN14, primers pairs 9873 & 9874 and 9875 & 9876 for *Se*CEN3, primers pairs 8451 & 8452 and 8453 & 8454 for *Se*CEN6, primers pairs 9877 & 9878 and 9879 & 9880 for *Se*CEN8, primers pairs 9881 & 9882 and 10090 & 10091 for *Se*CEN10, primers pairs 9885 & 9886 and 9887 & 9888 for *Se*CEN12, primers pairs 9889 & 9890 and 9891 & 9892 for *Se*CEN14 and primers pairs 11044 & 11045 and 11046 & 11047 for *Se*CEN15/8. The *amdS-GAL1*_p_ construct was amplified from pART001 using primers 8439 and 8440 and the plasmid backbone was amplified from pART001 using primers 8442 and 8441. Plasmids pART002-pART012 were constructed using the NEBuilder^®^ HiFi DNA Assembly method with the amplified backbone, the amplified *amdS-GAL1*p construct and of the two homology arms for the targeted centromere (Figure 1A).

**TABLE 2:** Plasmids used in this study.

Plamid	Relevant genotype	References
pUG-amdSYM	*amdS*	Solis-Escalante et al., 2013
pAG426GAL	*bla*, 2μ, *URA3, GAL1_p_*	Alberti et al., 2007
pART001	*bla*, 2μ, *amdS-GAL1_p_*	This study
pART002	*bla*, 2μ, *ScCEN8*-*amdS-GAL1_p_-Sc*CEN8	This study
pART003	*bla* 2μ, *ScCEN9*-*amdS-GAL1_p_ -Sc*CEN9	This study
pART004	*bla*, 2μ, *ScCEN10*-*amdS- GAL1_p_*	This study
pART005	*bla*, 2μ, *ScCEN12*-*amdS-GAL1_p_ -Sc*CEN12	This study
pART006	*bla*, 2μ, *ScCEN14*-*amdS-GAL1_p_ -Sc*CEN14	This study
pART007	*bla*, 2μ, *SeCEN3*-*amdS-GAL1_p_ Se*CEN3	This study
pART008	*bla*, 2μ, *SeCEN6*-*amdS-GAL1_p_ -Se*CEN6	This study
pART009	*bla*, 2μ, *SeCEN8*-*amdS-GAL1_p_ -Se*CEN8	This study
pART010	*bla*, 2μ, *SeCEN10*-*amdS-GAL1_p_ -Se*CEN10	This study
pART011	*bla*, 2μ, *SeCEN12*-*amdS-GAL1_p_ -Se*CEN12	This study
pART012	*bla*, 2μ, *SeCEN14*-*amdS-GAL1_p_ -Se*CEN14	This study

### Strain Construction

The *amdS-GAL1*_p_ integration cassettes targeting different centromeres were amplified from pART002 using primers 9857 and 9860, from pART003 using primers 8443 and 8510, from pART004 using primers 10088 and 9864, from pART005 using primers 9865 and 9868, from pART006 using primers 9869 and 9872, from pART007 using primers 9873 and 9876, from pART008 using primers 8451 and 8454, from pART009 using primers 9877 and 9880, from pART010 using primers 9881 and 10091, from pART011 using primers 9885 and 9888 and from pART012 using primers 9889 and 9893 (Figure 1B). Yeast transformation was done by electroporation as previously described (Thompson et al., 1998). A frozen stock of CBS 1483 was used to inoculate a 500 mL shake-flask containing 100 mL YPD. Upon exponential growth, the pre-culture was used to inoculate a fresh 500 mL shake-flask containing 100 mL YPD at a density of 5 × 10^6^ cells/mL, that was incubated at 20°C and 200 rpm until a density of 10^8^ cells/mL was reached. 50 mL of culture was re-suspended twice in 25 mL of ice-cold demi-water, re-suspended once in 2 mL 1 M ice-cold sorbitol, re-suspended once in 2 mL ice-cold 100 mM Lithium acetate with 10 mM dithiothreitol, re-suspended once in 2 mL ice-cold sorbitol and once in 250 μL ice-cold sorbitol. 50 μL of competent cells and up to 5 μL DNA were then electroporated in an ice-cold 0.2 cm cuvette with a pulse at 1.5 kV. Transformed cells were incubated in 0.5 mL YPD during 1 h, after which they were re-suspended in 100 μL of sterile demi-water and plated on selective medium. Strains were re-streaked from the transformation plates twice prior to storage of single colony isolates. Strain IMI350 (*Se*CEN6:*amdS*-*GAL1*_p_) was constructed by transforming CBS 1483 with 1 μg of insertion cassette amplified from pART008 (Figure 1B), and streaking on SM-Ac plates. Similarly, IMI353 was made using pART003, IMI359 was made using pART004, IMI360 was made using pART005, IMI361 was made using pART006, IMI363 was made using pART007, IMI366 was made using pART009, IMI367 was made using pART010, IMI368 was made using pART011, IMI369 was made using pART012. Strain IMI351 (*Sc*CHRV^–1^ *Sc*CHRX-*Se*CHRX^+1^ *Se*CHRVI^–^^1^ *Se*CHRX*-Sc*CHRX^–1^) was constructed by incubating strain IMI350 (*Se*CEN6:amdS-*GAL1*_p_) on YPGal medium and streaking on SMD-FAc plates. Similarly, IMI353 was constructed from IMI352, IMI373 from IMI359, IMI374 from IMI360, IMI375 from IMI361, IMI377 from IMI363, IMI380 from IMI366, IMI381 from IMI367, IMI382 from IMI368, and IMI383 from IMI369.

### Characterisation of Growth in Wort

Growth was characterized in triplicate in 100 mL serum bottles containing 100 mL of filtered undiluted industrial wort, supplemented with 1.6 mL/L of Pluronic PE 6100 antifoam (BASF, Ludwigshafen, Germany). Bottles were inoculated to an OD_660_ of 0.2 from pre-cultures grown for 2 days at 20°C in aerated 50 mL Greiner tubes on YPD, and incubated at 12°C at 200 RPM. Growth was monitored by OD_660_ and extracellular metabolites were measured by HPLC and GC analysis.

### Evaluation of the Mutagenic Effect on CBS 1483 of Electroporation and of Restreaking

To evaluate if electroporation could be responsible for the extensive chromosome CNV observed in strains in which chromosome copy removal was attempted, CBS 1483 was electroporated as described above without adding any DNA and streaked on YPD instead of selective medium. After restreaking twice on YPD, single colony isolates were made and named IMS0705-IMS0709 and sent for whole genome sequencing.

To evaluate if inherent instability of CBS 1483 could be responsible for the extensive chromosome CNV observed in strains in which chromosome copy removal was attempted, CBS 1483 was cultured on liquid YPD and streaked three times on YPD plates: a frozen stock of CBS 1483 was used to inoculate a 500 mL shake-flask containing 100 mL YPD. Upon exponential growth, the pre-culture was used to inoculate a fresh 500 mL shake-flask containing 100 mL YPD at a density of 5 x 10^6^ cells/mL, that was incubated at 20°C and 200 rpm until a density of 10^8^ cells/mL was reached. Medium was streaked on YPD and re-streaked twice to obtain single colony isolates, named IMS0710-IMS0714, that were sent for whole genome sequencing.

### Galactose-Promoter-Mediated Induction of Chromosome Missegregation and Selection of Ethanol Tolerant Mutants

Strain IMI361 (*Sc*CEN14*::amdS-GAL1_p_*) was grown overnight in 100 mL SMGAL in a 500 mL shake flask to induce chromosome missegregation and then transferred in SMD-FAC culture inoculated at an OD_660_ of 0.2 and incubated for 3 days at 20°C and 200 rpm. The mutagenized culture had an OD_660_ of 9.25 and 5 mL was used to inoculate two duplicate repeated batch fermentations in Multifors 2 Mini Fermenters (INFORS HT, Velp, Netherlands). Each batch was performed in 100 mL of SMD with 10% v/v ethanol, supplemented with 10 mg/L ergosterol, 420 mg/L Tween 80 and 0.9 mL/L antifoam C (Sigma Aldrich). The fermenters were kept at 20°C, stirred at 500 rpm, sparged with 50 mL/min N_2_ and the pH was maintained at 7 by automated addition of 2 M KOH. The CO_2_ composition in the offgas was analyzed using a BCP-CO_2_ gas analyser (Bluesens, Herten, Germany) and when the CO_2_ concentration dropped to less than 10% of its maximum during the batch, the fermenter was emptied leaving approximately 6 mL to inoculate the next batch, and fresh medium was added up to a total volume of 100 mL. The fermentation was monitored using IRIS software (version 6, Infors AG, Bottmingen, Switzerland) and samples of approximately 5 mL were taken at regular intervals to monitor viability using the FACS and to analyze metabolite concentrations by HPLC. At the end of the third batch, single colony isolates were obtained using FACS and restreaking, yielding strains IMX1875-IMX1890 for reactor GAL1 and strains IMX1891-IMX1894 for reactor GAL2.

### Chemical Induction of Chromosome Missegregation and Selection of Ethanol Tolerant Mutants

*Saccharomyces pastorianus* CBS 1483 was grown overnight in 100 mL YPD in a 500 mL shake flask, and transferred to 100 mL SMG containing 10 μg/mL of the mitotic inhibitor MBC (methyl benzimidazole-2-yl carbamate, Sigma Aldrich). After 2 days at 20°C and 250 rpm, approximately 2 mL of culture with an OD_660_ of 4.12 was used to inoculate two duplicate repeated batch fermentations in Multifors 2 Mini Fermenters (INFORS HT, Velp, Netherlands). Each batch was performed in 100 mL of SMG with 10% v/v ethanol, supplemented with 10 mg/L ergosterol, 420 mg/L Tween 80 and 0.9 mL/L antifoam C (Sigma Aldrich). The fermenters were kept at 20°C, stirred at 500 rpm, sparged with 50 mL/min N_2_ and the pH was maintained at 7 by automated addition of 2M KOH. The CO_2_ composition in the offgas was analyzed using a BCP-CO_2_ gas analyser (Bluesens, Herten, Germany) and when the CO_2_ concentration dropped to less than 10% of its maximum during the batch, the fermenter was emptied leaving approximately 6 mL to inoculate the next batch, and fresh medium was added up to a total volume of 100 mL. The fermentations were performed as described in the previous paragraph. At the end of the third batch, single colony isolates were obtained using FACS and re-streaking, yielding strains IMS687-IMS704 for reactor MBC1 and strains IMS715-IMS720 for reactor MBC2.

### FACS Analysis and Sorting

Cultures for FACS analysis and sorting were diluted in sterile Isoton II and vortexed thoroughly to disrupt cell aggregates. For cell sorting, 50 mM EDTA was added to disrupt cell aggregates formed by flocculation. The cultures were analyzed on a BD FACSAriaTM II SORP Cell Sorter (BD Biosciences, Franklin Lakes, NJ, United States) equipped with 355, 445, 488, 561, and 640 nm lasers and a 70 μm nozzle, and FACSFlowTM sheath fluid (BD Biosciences). Correct cytometer performance was evaluated prior to each experiment by running a Cytometer Setup and Tracking cycle using a CS&T bead kit (BD Biosciences) for calibration. Drop delay for sorting was determined by running an Auto Drop Delay cycle using Accudrop Beads (BD Biosciences). Morphology of the cells was analyzed by plotting forward scatter (FSC) against side scatter (SSC). Prior to sorting, at least 10^5^ events were analyzed. Sorting regions (“gates”) were set on these plots to determine the types of cells to be sorted. Gated single cells were sorted in 96-well microtiter plates containing YPD using a “single cell” sorting mask (0/32/16), and the plates were incubated at RT for 2 days. FACS data were analyzed using FlowJo^®^ software (version 3.05230, FlowJo, LLC, Ashland, OR, United States) (Gorter de Vries et al., 2019a).

### Determination of the Fraction of Growing Cells

After FACS sorting, the fraction of growing cells was determined by counting the number of wells in which growth was observed. For populations with low viabilities, up to 1000 cells were sorted per well and Poisson statistics were used to estimate the fraction of growing cells (Dube et al., 2008). The fraction of growing cells was calculated from (*P*), the fraction of wells containing a colony, (*W*) the total number of wells and (*n*), the total number of cells sorted into the wells (Eq. 1).

(1) $$\mathrm{Fraction}\,\mathrm{of}\,\mathrm{growing}\,\mathrm{cells}=\frac{\ln\left(1-P\right)•W}{n}$$

### Screening of Galactose-Promoter Mutagenized Isolates With Increased Ethanol Tolerance

Isolates IMX1875-IMX1890 from reactor GAL1 and isolates IMX1891-IMX1894 from reactor GAL2 were screened for increased ethanol tolerance by evaluating growth on SMG with 10% ethanol v/v in airlock-capped bottles. Precultures of the isolates, of unmutagenized CBS 1483 and of unmutagenized IMI361 were grown at 20°C and 200 rpm in 500 ml shake flasks containing 100 mL SMG for 5 days. After washing of the precultured cells in demineralized water, airlock-capped 100 mL cylindrical bottles containing 100 mL SMG with 10% ethanol v/v were inoculated to an OD_660_ of 1. The bottles were incubated at 20°C and 200 rpm during 8 days and regularly sampled through the septum using a needle to measure OD_660_ and extracellular metabolite concentrations.

### Screening of MBC-Mutagenized Isolates With Increased Ethanol Tolerance

Isolates IMS0687-IMS0704 from reactor MBC1 and isolates IMS0715-IMS0720 from reactor MBC2 were screened for increased ethanol tolerance by evaluating growth on SMG with 10% ethanol v/v. Precultures of the isolates and unmutagenized CBS 1483 were grown at 20°C and 200 rpm in 100 mL SMG in 500 mL shake flasks for 7 days. 500 mL shake flasks containing 100 mL SMG with 10% ethanol v/v were inoculated from these precultures at an OD_660_ of 0.5 and incubated at 20°C and 200 rpm during 142 h. The OD_660_ and extracellular metabolite concentrations were measure at regular intervals to monitor growth.

### Characterisation of Ethanol Tolerance Under Micro-Aerobic Conditions

The ethanol tolerance of galactose-promotor-mutagenized isolates IMX1882, IMX1886, IMX1891, and IMX1893, and of MBC-mutagenized isolates IMS0687, IMS0698, IMS0699, IMS0703, and IMS0716 was characterized under micro-aerobic conditions, by evaluating growth in SMG with 10% ethanol in airlock-capped bottles. Precultures of the isolates, of unmutagenized CBS 1483 and of unmutagenized IMI361 were grown at 20°C and 200 rpm in 500 ml shake flasks containing 100 mL SMG for 5 days. As isolate IMS0699 did not grow to as sufficient OD_660_, it was discarded for the rest of the experiment. After washing of the precultured cells in demineralized water, triplicate airlock-capped 250 mL cylindrical bottles containing 100 mL SMG with 10% ethanol v/v supplemented with 10 mg/L ergosterol and 420 mg/L Tween 80 were inoculated to an OD_660_ of 1. The bottles were incubated at 20°C and 200 rpm during 4 days and regularly sampled through the septum using a needle to measure OD_660_ and extracellular metabolite concentrations.

## Results

### Engineering Chromosome Copy Number in *S. pastorianus* Type Strain CBS 1483

**FIGURE 2:**
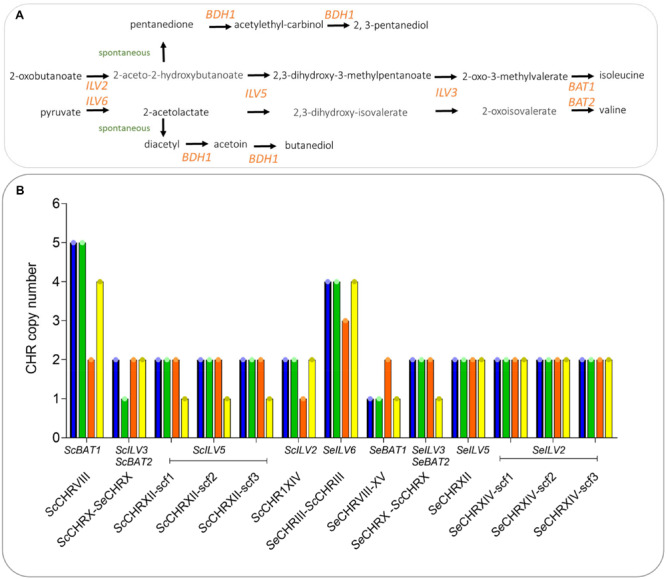
Vicinal diketones formation in *S. pastorianus*. **(A)** Schematic illustration of the diacetyl, pentanedione and branched chain amino acid biosynthesis pathways. **(B)** Valine, isoleucine, pentanedione and diacetyl biosynthetic pathways and chromosome copy number of the genes involved in the metabolic routes in strains CBS 1384 (blue bar), IMI373 (ScCHRX-SeCHRX^– 1^ SeCHRIX^–1^) (green bar) that was targeted for loss of ScCHRX, IMI375 (*Sc*CHRII^–1^ *Sc*CHRV^–1^ *Sc*CHRVIII^–3^ *Sc*CHRXIII^–1^ ScCHRXIV^–1^ SeCHRI^–1^ *Se*CHRIII-*Sc*CHRIII^–1^ *Se*CHRVII-*Sc*CHRVII^–1^ *Se*CHRVIII-XV^+1^) (orange bar) that was targeted for loss of ScCHRXIV and IMI381 (*Sc*CHRIV^+1^ *Sc*CHRVIII^–1^ *Sc*CHRIX^+1^ *Sc*CHRXI^–1^ *Sc*CHRXII^–1^ *Se*CHRX-*Se*CHRX^–1^ *Se*CHRII-IV^–1^ *Se*CHRXI^+1^) (yellow bar) that was targeted for the loss of SeCHRX. The chromosome copy number was predicted using Magnolya (Nijkamp et al., 2012).

**FIGURE 3:**
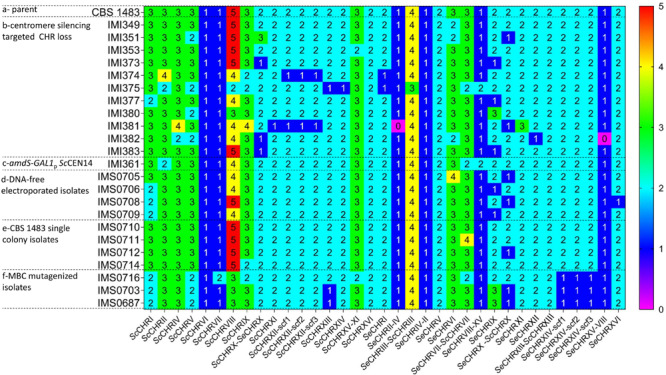
Chromosome copy number in various *S. pastorianus* strains. **(a)** Chromosome copy number of the parental *S. pastorianus* strain CBS 1483. **(b)** Chromosome copy number of strains obtained after centromere silencing of *Se*CEN6 (in IMI350), *Sc*CEN9 (in IMI352), *Sc*CEN10 (in IMI359), *Sc*CEN12 (in IMI360), *Sc*CEN14 (in IMI361), *Se*CEN3 (in IMI363), *Se*CEN8 (in IMI366), *Se*CEN10 (in IMI367), *Se*CEN12 (in IMI368) and *Se*CEN14 (in IMI369). **(c)** Chromosome copy number of a strain engineered for targeted loss of ScCEN14 before centromere silencing induction. **(d)** Chromosome copy number of strains after electroporation without DNA. **(e)** Chromosome copy number of single isolates of the *S. pastorianus* strain CBS 1483. **(f)** Chromosome copy number of isolates obtained of treatment with methyl benzimidazole 2 yl carbamate (MBC). Chromosome copy number was predicted using Magnolya (Nijkamp et al., 2012).

In order to assess the phenotypic impact of chromosome CNV in an alloaneuploid *S. pastorianus* genome, we attempted to delete copies of individual chromosomes in strain CBS 1483 (Bolat et al., 2013; Brickwedde et al., 2017). Due to the hypothesized role of their CCNV in the production of off-flavor diacetyl, chromosomes harboring genes of the valine biosynthesis pathway were targeted (van den Broek et al., 2015): chromosomes *Se*CHRIII (*SeILV6*), *Se*CHRVI (negative control), *Se*CHRVIII (*SeBAT1*), *Se*CHRX (*SeILV3* and *SeBAT2*), *Se*CHRXII (*SeILV5*), *Se*CHRXIV (*SeILV2*), *Sc*CHRVIII (Sc*BAT1*), *Sc*CHRIX (negative control), *Sc*CHRX (*ScILV3* and *ScBAT2*), *Sc*CHRXII (*ScILV5*), and *Sc*CHRXIV (*ScILV2*) (Figure 2A). In *S. cerevisiae*, cloning of the *GAL1* promoter and *URA3* marker adjacent to a centromere sequence enabled targeted loss or gain of specific chromosomes (Reid et al., 2008; Anders et al., 2009). In contrast to *URA3* that needs to be used in an auxotrophic host, the *amdS* marker can be selected for by growth with acetamide as sole nitrogen source and similarly, to *URA3* can be counter-selected for by growth in the presence of fluoroacetamide, but in any strains including prototrophs (Solis-Escalante et al., 2013). Therefore, plasmid pART001 containing a centromere-silencing cassette with the *amdS* marker upstream of *GAL1*_p_ was constructed. In order to insert this cassette in targeted chromosomes, 1000 bp of genetic material was amplified from both sides of each targeted integration site, immediately downstream of the centromere of targeted chromosomes (Figure 1). The amplified homology arms were inserted into pART001 at the flanks of the *amdS-GAL1_p_* cassette, resulting in plasmids pART002 to pART012. CBS 1483 was then transformed with PCR-amplified insertion cassettes from pART002 to pART012 and successful transformants were selected on SMD-AC medium. After verification of correct insertion by PCR-amplification of the targeted *CEN* locus, single colony isolates were stocked as IMI349 and IMI350 (*Se*CEN6), IMI352 (*Sc*CEN9), IMI359 (*Sc*CEN10) IMI360 (*Sc*CEN12), IMI361 (*Sc*CEN14), IMI363 (*Se*CEN3), IMI366 (*Se*CEN8), IMI367 (*Se*CEN10), IMI368 (*Se*CEN12), and IMI369 (*Se*CEN14) (Table 1). The strains harboring *amdS-GAL1*_p_ centromere silencing cassettes were then grown on YPGal medium, to induce centromere silencing and therefore chromosome missegregation by growth on galactose. Single colony isolates were purified and stocked as IMI349 and IMI351 (*Se*CHRVI), IMI353 (*Sc*CHRIX), IMI373 (*Sc*CHRX), IMI374 (*Sc*CHRXII), IMI375 (*Sc*CHRXIV), IMI377 (*Se*CHRIII), IMI380 (*Se*CHRVIII), IMI381 (*Se*CHRX), IMI382 (*Se*CHRXII), and IMI383 (*Se*CHRXIV) (Table 1). These isolates were whole genome sequenced, along with the parental strain CBS 1483. Chromosome copy number was determined by analyzing sequencing coverage (Nijkamp et al., 2012). Single chromosome copies were successfully deleted when targeting *Sc*CHRVI, *Sc*CHRX-*Se*CHRX, *Sc*CHRXII, *Sc*CHRXIV, *Se*CHRX-*Sc*CHRX and *Se*CHRXII but not when targeting *Se*CHRIII-*Sc*CHRIII, *Se*CHRVIII-XV and *Se*CHRXIV indicating successful removal of the targeted chromosome in 71% of the constructed strains (Table 1). However, coverage analysis also revealed alterations of the copy number of non-targeted chromosomes in all but one [IMI349 (*Se*CHRVI)] of the tested isolates. The magnitude of the CCNV varied from 1 to 10 chromosomes (Figure 3). The most extreme case was illustrated by the isolate IMI375 that had lost 11 chromosome copies and gained one, that resulted in a CCNV of ten chromosomes. To investigate if galactose-induced centromere silencing was responsible for the untargeted CCNV, non-induced intermediate strain IMI361 (*Sc*CEN14::*amdS*-*GAL1*_p_) was sequenced as well. IMI361 displayed an increased copy number of *Se*CHRVIII-XV and decreased copy number of *Sc*CHRVIII and chimeric *Se*CHRVII-*Sc*CHRVII (+1 or −1 copy each) relative to untransformed CBS 1483, indicating that the insertion of the *amdS-GAL1*_p_ cassette itself may already cause CCNV. However, the IMI361-derived strain IMI375 (Δ*Sc*CEN14), displayed additional CCNV. Expectedly IMI375 harbored one copy less of the targeted *Sc*CHRXIV (−1, this will be denoted as *Sc*CHRXIV^–1^ throughout the manuscript), it also showed decreased copy number for six additional chromosomes (*Sc*CHRV^–1^ *Sc*CHRVIII^–2^ *Sc*CHRIX^–1^ *Sc*CHRXIII^–1^ *Se*CHRI^–1^ *Se*CHRIII-*Sc*CHRIII^–1^) relative to IMI361, indicating that induction the *amdS-GAL1*_p_ cassette also contributed to the modification of genotype (Figure 3).

### Genetic Instability of CBS 1483 and Mutagenic Effect of Electroporation

The observation that CCNV occurred when inserting the *amdS-GAL1*_p_ cassette could be the result of an inherent instability of CBS 1483 or more generally alloaneuploid *S. pastorianus* strains, to a mutagenic effect of the general transformation procedure or to a specific effect of insertion of *amdS-GAL1*_p_. To investigate the stability of CBS 1483, a frozen aliquot was grown in YPD medium for two generations as this would be done for a transformation, then the culture was streaked on a YPD plate and five randomly selected single colony isolates were re-streaked on two successive YPD plates to simulate isolation procedure. The resulting strains were stocked as IMS0710-IMS0714. In parallel, cells from the same YPD culture were prepared for transformation and electroporated in absence of DNA. The resulting strains were plated on YPD and five randomly picked single colony isolates were re-streaked on two successive YPD plates. The resulting strains were stocked as IMS0705-IMS0709. Eight of these isolates IMS0705-IMS0706, IMS0708-IMS709, and IMS0710-IMS0712, IMS0714 were sequenced and chromosome copy number was determined by analyzing sequencing coverage and comparing the copy numbers to those of CBS 1483. Non-electroporated cell lines IMS0710-IMS0712, IMS0714 already exhibited moderate CCNV, out the four sequenced isolates three showed gain or loss of a single chromosome. The cell line IMS0711 gained one copy of chimeric *Se*CHRVII-*Sc*CHRVII, while IMS0712 and IMS0714 has lost one copy of *Se*CHRX-ScCHRX and *Sc*CHRIX, respectively. The fourth sequenced isolate IMS0710 showed a chromosome complement identical to that of CBS 1483 (Salazar et al., 2019). Overall, the non-electroporated single cell lines have an average of 76.8 ± 0.8 chromosomes which represent an average loss of 0.2 chromosome relative to CBS 1483 whose genome comprise 77 chromosomes (Figure 3). Upon DNA-free electroporation, karyotype of single cell lines IMS0705-IMS0706, IMS0708-IMS709 was more significantly affected as more chromosomes displayed CCNV. With the exception of the *Se*CHRVI in IMS0705 that showed an increase from three to four copies, all other CCNV involved loss of a single copy. Some chromosomes were affected in several strains, the copy number of *Sc*CRHVIII and *Sc*CHRI was decreased in three isolates (IMS0705, IMS0706, and IMS0709). The chromosome number of *Se*CHRX-*Sc*CHRX was decreased in two of the sequenced cell lines (IMS0705 and IMS0708). Thus on average electroporated single cell lines had 74.5 ± 1.0 chromosomes which represent an average loss of 2.5 chromosome relative to CBS 1483. These results indicated that the chromosome copy number of CBS 1483 is inherently unstable under cultivation conditions, and that the procedure of electroporation significantly exacerbates copy number alterations (^Student *t*–test^*p*_value_ = 1.1E-2). It should be noted that the strains in which copy number alterations were attempted showed even higher CCNV, with an average of 73.4 ± 3.8 chromosomes (Figure 3).

### Mutants With Altered Chromosome Copy Number Display Diverse Phenotypes

**FIGURE 4:**
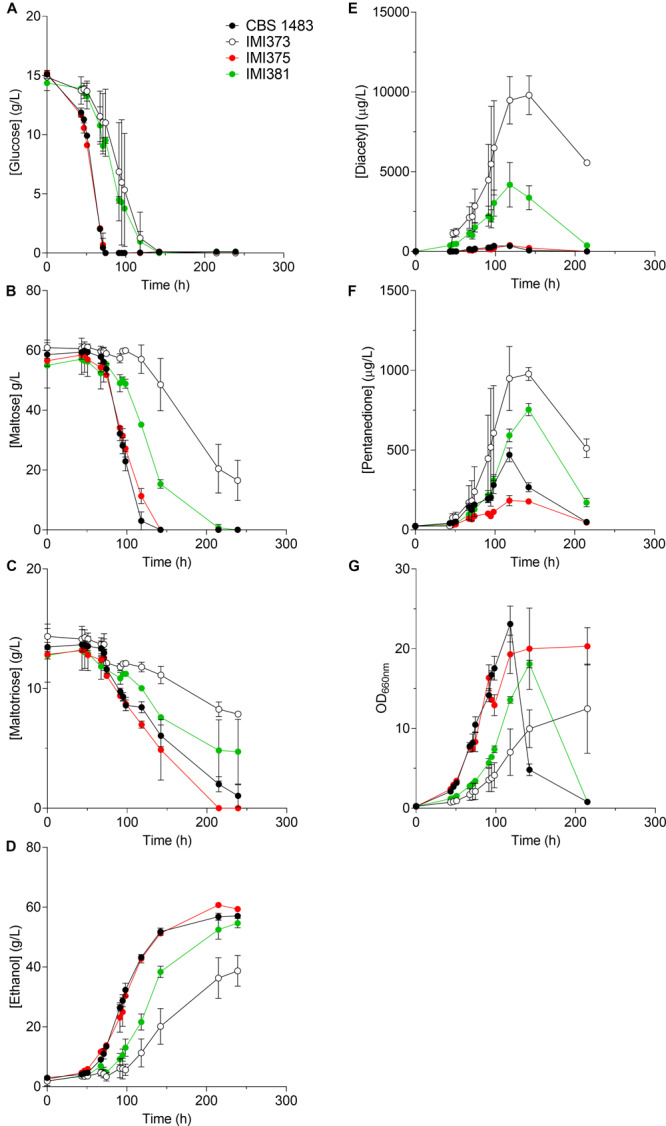
Characterisation of CBS 1483, and IMI373, IMI375 and IMI381 under brewing conditions. The *S. pastorianus* strains CBS 1483 (black circle), IMI373 (ScCHRX-SeCHRX^–1^ SeCHRIX^–1^) (white circle), IMI375 (*Sc*CHRII^–1^ *Sc*CHRV^–1^ *Sc*CHRVIII^–3^ *Sc*CHRXIII^–1^ ScCHRXIV^–1^ SeCHRI^–1^ *Se*CHRIII-*Sc*CHRIII^–1^ *Se*CHRVII-*Sc*CHRVII^–1^ *Se*CHRVIII-XV^+1^) (red circle) and IMI381 (*Sc*CHRIV^+1^ *Sc*CHRVIII^–1^ *Sc*CHRIX^+1^ *Sc*CHRXI^–1^ *Sc*CHRXII^–1^ *Se*CHRX-*Se*CHRX^–1^ *Se*CHRII-IV^–1^ *Se*CHRXI^+1^) (green circle) were grown in air-capped 100 ml serum bottles in undiluted industrial wort at 12 °C. Average and standard deviation from duplicates (IMI373 and IMI375) or triplicates (IMI381 and CBS 1483) are shown. **(A)** displays glucose, **(B)** maltose, **(C)** maltotriose, **(D)** ethanol determined by liquid chromatography; **(E)** diacetyl, **(F)** pentanedione concentrations produced in industrial wort measured using static headspace gas chromatography and **(G)** optical density measured at 660 nm (OD_660_ is directly related with biomass concentration in suspension).

The presence of untargeted chromosome copy number alterations prevented the initially-intended investigation of the effect of specific copy number changes on diacetyl production. However, it resulted in a set of isogenic strains with extensive CCNV. Since CCNV can result in altered phenotypes of potential interest for industrial application (Gorter de Vries et al., 2017), we characterized three isolates by growing them in industrial wort under micro-aerobic conditions. IMI373 (*Se*CHRX-*Sc*CHRX^–1^ *Se*CHRIX^–1^), IMI375 (*Sc*CHRII^–1^ *Sc*CHRV^–1^ *Sc*CHRVIII^–3^ *Sc*CHRIX^1^ *Sc*CHRXIII^–1^ *Sc*CHRXIV^–1^ *Se*CHRI^–1^ *Se*CHRIII-*Sc*CHRIII^–1^ *Se*CHRVII-*Sc*CHRVII^–1^ SeCHRVIII-XV^+1^) and IMI381 (*Sc*CHRIV^+1^ *Sc*CHRVIII^–1^ *Sc*CHRIX^+1^ *Sc*CHRXI^–1^ *Sc*CHRXII^–1^ *Se*CHRII-IV^–1(D)^ *Se*CHRX-*Sc*CHRX^–1^ *Se*CHRXI^+1^) were characterized by monitoring their growth rates, sugar consumption profiles and diacetyl production (Figure 4). The average growth rates of IMI373, IMI375 and IMI381 were 0.031, 0.040, and 0.033 1/h, respectively, representing an up to 38% decrease relative to the growth rate of 0.050 1/h of CBS 1483. In addition, IMI373 and IMI375 did not flocculate as strongly as CBS 1483: the OD_660_ decreased by 90 and 95% relative to its maximal value after 200 h of fermentation for CBS 1483 and IMI381, respectively, while it only decreased by 20% for IMI373 and did not decrease at all for IMI375 (Figure 4). While sugar consumption profiles cannot be compared directly due to the differences in growth rates, IMI373 and IMI381 clearly did not consume all di- and tri-glucosides (Figure 4), a trait that was not correlated with the flocculation phenotype of the strain. In accordance with the hypothesized effect of copy number differences of chromosomes harboring genes of the valine synthesis pathway (van den Broek et al., 2015), IMI373 and IMI381 displayed altered diacetyl production profiles. While the concentration of diacetyl did not exceed 400 μg/l for CBS 1483 and IMI375, concentrations of about 4 and 10 mg/L were reached for IMI381 and IMI373, respectively. Moreover, while diacetyl concentrations decreased to 300 μg/L for IMI381, they remained above 5 mg/L for IMI373 (Figure 4). In both cases these levels were above the diacetyl sensory threshold that is fixed at 150 μg/L in lager fermented products (Krogerus and Gibson, 2013). Although pentanedione does not contribute to off-flavor formation, the production-re-consumption profiles correlated with those of diacetyl (Figure 4). The IMI373 and IMI381 phenotypes were characterized by significant diacetyl accumulation (Figure 4) could be associated with a decreased flux through the branched chain amino acid pathway and be linked to the loss of single *Sc*CHRX-*Se*CHRX and dual *Sc*CHRXII and *Se*CHRX-*Sc*CHRX, respectively (Figure 2B). These chromosomes carries genes *ILV5* (CHRXII) and *ILV3* (CHRX) that act downstream 2-acetolactate, precursor of diacetyl. Overall, these results indicated a strong phenotypic impact of chromosome copy number alterations, notably affecting the industrially-relevant traits of flocculation, sugar utilization and diacetyl production.

### Centromere Silencing as a Strain Engineering Tool

The impact of centromere-silencing on CCNV, might thus be used as a mutagenesis instrument that could result in new phenotypes of industrial interest. Recent trends for high gravity beer brewing result in inhibition due to increasing ethanol concentrations (Puligundla et al., 2011). Therefore, the methods of centromere silencing-induced CCNV was evaluated to improve ethanol tolerance of *S. pastorianus* CBS 1483. To this end, IMI361 (*amdS-GAL1*_p_ cassette in *Sc*CHRXIV) was grown on YPGal medium to induce centromere silencing. The mutagenized population was inoculated in duplicate bioreactors (GAL1 and GAL2) containing SMD medium with 10% ethanol v/v supplemented with anaerobic growth factors. Growth was monitored by measuring the off-gas CO_2_ concentration and the medium was replenished when growth was completed, this was perpetuated over four sequential batches. Due to sparging with N_2_ gas, the concentration of ethanol decreased steadily during the batches until growth could occur. The viability dropped below 40% in all batches, indicating a strong inhibitory selective effect of ethanol. During the fourth batch, samples were taken and single cell lines named IMX1875-IMX1889 were isolated from reactor GAL1 and IMX1890-IMX1893 from reactor GAL2.

**FIGURE 5:**
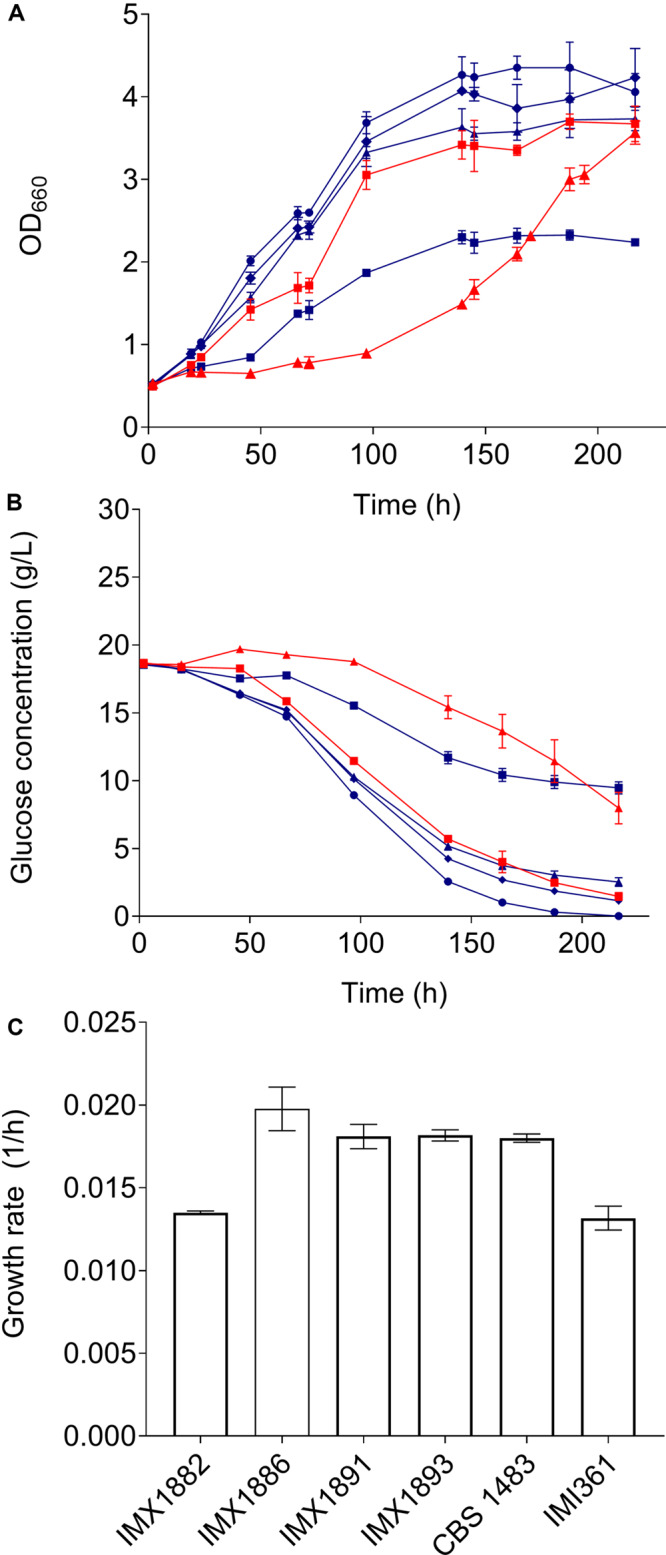
Characterisation of mutants obtained by centromere-silencing mutagenesis in medium containing 10% ethanol. Strains IMX1882 (blue squares), IMX1886 (blue triangles), IMX1891 (blue diamonds), IMX1893 (blue circles), CBS 1483 (red squares) and IMI361 (red triangles) were grown in triplicate in micro-aerobic bottle fermentations on SMD medium containing 10% ethanol at 20°C during 9 days. During the experiment OD_660_ **(A)** and extracellular glucose concentrations **(B)** were monitored. The growth rates were calculated from at least 6 measurements with an *R*^2^ superior to 0.95 **(C)**.

The ethanol tolerance of CBS 1483, IMI361 and IMX1875-IMX1893 was evaluated by growing them in airlock-capped bottles containing 100 mL SMD with 10% ethanol v/v at 20°C for 9 days. Growth was monitored by measuring the OD_660_. CBS 1483 and IMI361 reached final OD_660_ values of 3.78 and 2.68. While fourteen of the isolates reached a higher OD_660_ than IMI361, only four mutants reached a higher OD_660_ than CBS 1483. The four single cell lines IMX1878, IMX1891, IMX1886, IMX1893 displayed a biomass yield at least 22 and 72% higher that of CBS 1483 and IMI361, respectively, and the isolate IMX1893 reached the highest OD values that were 50 and 110% higher that of CBS 1483 and IMI361, respectively. Based on their improved growth capacity under ethanol stress the mutants cell lines IMX1882, IMX1886, IMX1891 and IMX1893 were selected for further characterization. Together with CBS 1483 and IMI361, the four mutants were grown in triplicate in bottles as described above during 9 days, and samples were taken at regular intervals to measure the OD_660_ and extracellular metabolites. While the growth rates of mutant strains did not significantly exceed that of CBS 1483 (Figure 5), the OD_660_ of IMX1891 and IMX1893 was significantly higher than that of CBS 1483 throughout the whole culture (Figure 5). Correspondingly, glucose consumption was faster in IMX1891 and IMX1893 than in CBS 1483, and IMX1893 depleted all glucose after 216 h, while 1.5 g/L glucose was still left for CBS 1483 (Figure 5). These results indicate a moderate improvement of growth in the presence of ethanol for some of the obtained mutants.

### Chemical Induction of Chromosome Missegregation as a Strain Engineering Tool

Strains obtained by centromere-silencing displayed large CCNV and industrially-relevant phenotypic diversity. While mutagenesis using centromere-silencing and selection for ethanol tolerant mutants resulted mostly in mutants with inferior growth in the presence of ethanol, some isolates consistently outperformed their parental strain. While centromere-silencing may have potential as a mutagenesis method, its reliance on genome editing to introduce centromere-silencing cassettes makes the resulting strains genetically modified organisms. Chromosome missegregation can also be achieved by exposure to chemicals, such as the mitotic inhibitor methyl benzimidazole 2 yl carbamate (MBC) (Wood, 1982). Exposure to MBC has been successfully used as a mutagenesis method to obtain a bioethanol-producing *Saccharomyces cerevisiae* strain with improved fermentative capacity under high-gravity conditions, enhanced viability after drying, and higher final ethanol titers (Zheng et al., 2013, 2014; Zhang et al., 2015). Therefore, we mutagenized strain CBS 1483 by growing it in SMD medium containing 10 μg/mL MBC and selected ethanol-tolerant mutants by growth during four consecutive batches in duplicate reactors (MBC1 and MBC2) containing SMD medium with 10% ethanol (v/v) supplemented with anaerobic growth factors. During the fourth batch, samples were taken and strains IMS0687-IMS0704 were isolated from reactor MBC1 and strains IMS715-IMS720 from reactor MBC2.

**FIGURE 6:**
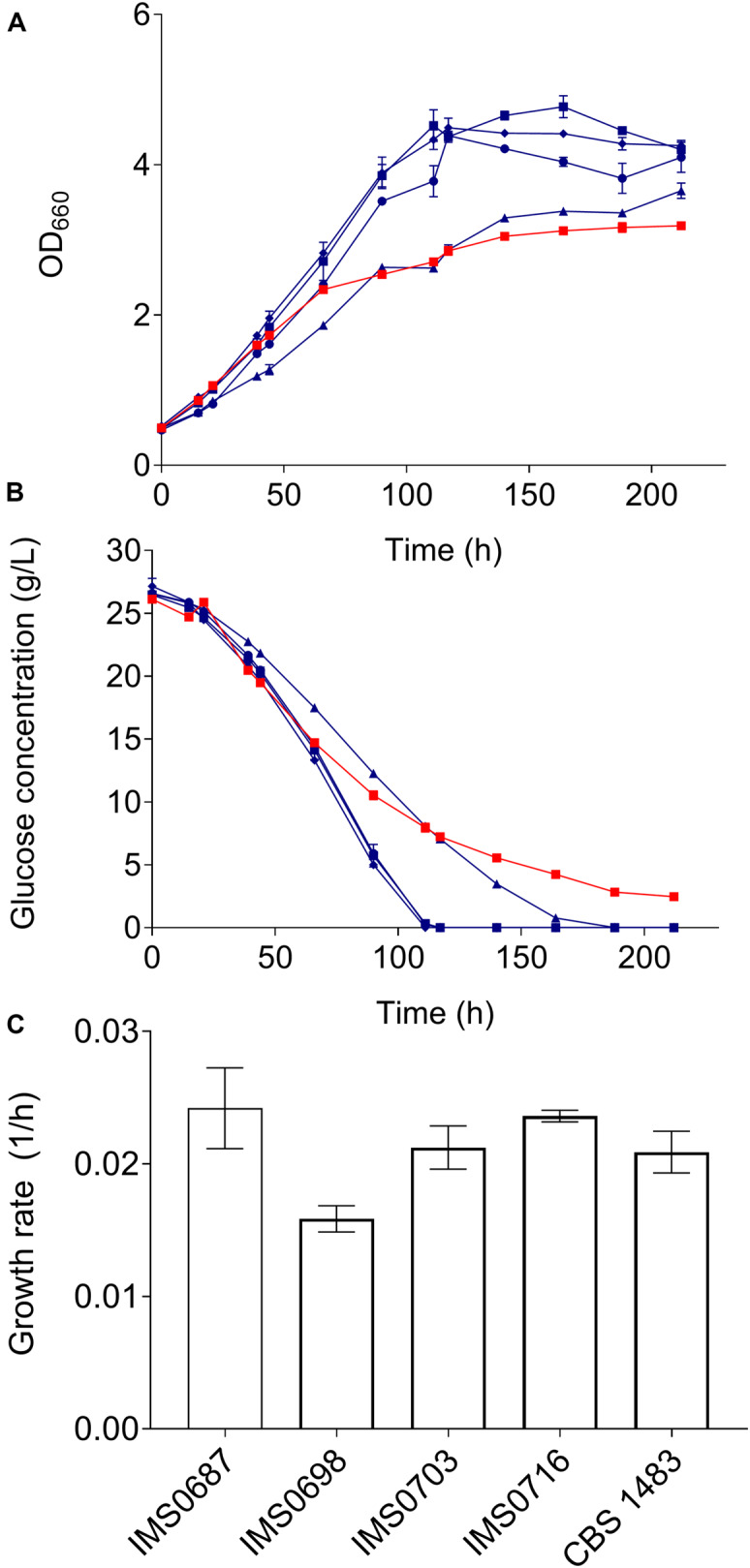
Characterisation of mutants obtained by MBC mutagenesis in medium containing 10% ethanol. Strains IMS0687 (blue squares), IMS0698 (blue triangles), IMS0703 (blue diamonds), IMS0716 (blue circles) and CBS 1483 (red squares) were grown in triplicate in micro-aerobic bottle fermentations on SMG medium containing 10% ethanol at 20°C during 9 days. During the experiment OD_660_ **(A)** and extracellular glucose concentrations **(B)** were monitored. The growth rates were calculated from at least 6 measurements with an *R*^2^ superior to 0.95 **(C)**.

The ethanol tolerance of CBS 1483, IMS0687-IMS0704 and IMS0715-IMS0720 was evaluated in batch cultures in shake flasks containing 100 mL SMD with 10% ethanol (v/v) at 20°C during 7 days. Growth was monitored by measuring the OD_660_ and extracellular metabolite concentrations. The strains IMS0687, IMS0698, IMS0703, and IMS0716 that reached the higher OD_660_ were selected and grown along the parental strain CBS 1483 in triplicate in bottles with SMD 10% ethanol as described above for 10 days. Samples were taken at regular intervals to measure the OD_660_ and extracellular metabolites. The exponential growth rates of IMS0687, IMS0703 and IMS0716 were similar to that of CBS 1483, and the growth rate of IMS0698 was significantly lower (Figure 6). However, after about 60 h the growth of CBS 1483 slowed down (Figure 6), but still reached OD_660_ of 3.2 after 212 h. In contrast IMS0687, IMS0703, and IMS0716 reached 3.2 after less than 90 h. Moreover, the final OD_660_ of the mutant strains was between 15 and 33% higher than that of CBS 1483. Correspondingly, IMS0687, IMS0703, and IMS0716 consumed all glucose within 111 h and IMS0698 within 164 h. In the same period the parental strain CBS 1483 only consumed 88% of available sugar (Figure 6). These results indicate that these mutants have acquired more robust growth and sugar utilization in the presence of 10% ethanol.

To evaluate the impact of MBC on the genome composition of improved mutants IMS0687, IMS0703, and IMS0716, these isolates were subsequently sequenced and chromosome copy number was determined by analyzing sequencing coverage (Nijkamp et al., 2012). The changes in genome composition were significantly larger than what had been previously observed for isolates obtained through centromere silencing (^student *t*–test^*p*_value_ = 2.0E-4). On average, MBC treated mutants exhibiting ethanol tolerance have lost and gained 10.7 ± 1.2 chromosomes resulting in genomes composed of 68.7 ± 0.6 chromosomes. Although the set of sequenced mutants was small, the strains IMS0687, IMS0703, and IMS0716 displayed unique genome composition. However they all showed systematic CCNV for ScCHRI^–1^, ScCHRV^–1^, ScCHRVIII^–1^, and SeCHRXIV^–1^.

**FIGURE 7:**
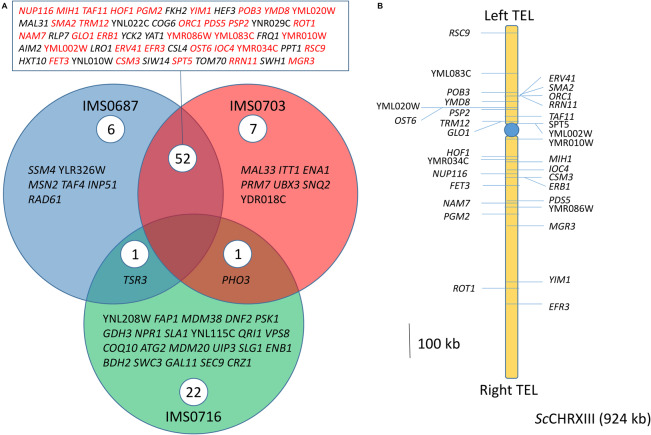
SNV on monosomic ScCHRXIII after MCB treatment predominantly originate from Loss of heterozygosity. **(A)** Venn Diagram of Single Nucleotide Variations (SNV) found in coding sequences relative to the parental strain CBS 1483 identified in *S. pastorianus* strains IMS0687 (blue), IMS0703 (red) and IMS0716 (green) obtained after MBC treatment. Genes found in IMS0687-IMS0703 intersection and denoted in red were located on *Sc*CHRXIII whose copy number decreased from two to one copy. **(B)** Schematic representation of the SNV derived from LOH on ScCHRXIII of IMS0687 and IMS0703 MBC treated mutants.

Chromosome copy number variation might not be the only genetic determinant underlying the ethanol tolerance, single nucleotide variations (SNV) and genetic reduction involving loss of heterozygosity might be as critical. However, current assembly algorithms reduce genome assemblies to consensus sequences. Information about sequence variation between different chromosome haplotypes is not captured by consensus assemblies (Salazar et al., 2019). In spite the awareness of this pitfall, analysis of sequence variation of IMS0687, IMS0703, and IMS0716 relative to the CBS 1483 consensus sequence was performed (Figure 3). While IMS0716 harbored variations in 24 genes, about 2.5-fold more genes were affected in strains IMS0687 and IMS0703. Out of the 59 and 60 genes identified carrying mutations in IMS0687 and IMS0703, respectively, 52 were shared by the two strains. A close inspection of these SNV revealed that 33 genes (64%) were located on *Sc*CHRXIII (Figure 7). The two mutant strains were monosomic for *Sc*CHRXIII conversely to the parental CBS 1483 that was disomic and this SNV enrichment on ScCHRXIII therefore reflected an heterozygosity of the two chromosomal copies in CBS 1483 and likely not occurrence of de novo mutations. This shows that in addition to impact on gene dosage, CCNV through loss of heterozygosity might also play an important role in the expression of alleles (likely recessive) necessary to improve ethanol tolerance phenotype.

## Discussion

*Saccharomyces pastorianus* is an interspecific hybrid of *S. cerevisiae* and *S. eubayanus* (Nakao et al., 2009; Libkind et al., 2011) that has been domesticated in Europe since the late Middle Ages (Meussdoerffer, 2009) and that accounts for 89% of brewed beer worldwide. Both Saaz and Frohberg type lager brewing yeast are characterized by an alloaneuploid genome. The present study demonstrated that these yeasts are intrinsically genetically instable and that this property could be harnessed to speed up strain improvement programs. While this notion was generally well accepted, we quantified the impact of this instability on the chromosome complement of the Frohberg *S. pastorianus* strain CBS 1483. It appeared that even on a limited set of tested isolates (#4) chromosomal copy number alterations were noticeable (Figure 3). Although limited to a single chromosome variation, this showed that over a small number of transfers new karyotypes can emerged. Next to replicative aging, such genetic drift might contribute to the deterioration of brewing properties of lager yeast after re-pitching, an industrial practice that consists in harvesting of the yeast biomass upon completion of fermentation and its reuse in subsequent fermentations (Jenkins et al., 2003). While decreased brewing performance was often associated to the strong selection pressure encountered by yeast in brewing environment (high ethanol and CO_2_, nutrient limitation and low temperature) (Gibson et al., 2007; Kalayu, 2019), our data showed that other factors such as natural genetic instability and physical treatment (e.g., electroporation) may strongly contribute to changes in karyotype. Poor genetic stability is a highly undesired trait at industrial brewing scale, since lager beer properties (e.g., flavor profiles, attenuation) are strictly standardized and contribute to the specific organoleptic signature of one beverage and of the strain that is associated to it.

This study confirmed that isolated variants from a strain population with CCNV exhibited distinct brewing related phenotypes as variation in flocculation, diacetyl reduction, sugar consumption rate and attenuation (Bolat et al., 2008; van den Broek et al., 2015). Therefore, methodologies to manipulate chromosome copy number represent a valuable strategy for lager yeast strain improvement (Gorter de Vries et al., 2017). Targeted engineering of chromosome copy number using centromere silencing was successfully applied to induce CCNV. But in contrast to previous examples in *S. cerevisiae* (Reid et al., 2008), this method unexpectedly yielded extensive off-targeting (Figure 3), which precluded the use of this approach to eliminate a single chromosome copy and therefore assess the impact of CCNV of an individual chromosome. Moreover, the requirement to introduce the centromere destabilizing construct further precludes the application of this method for the development of new brewing yeast primarily caused by producers’ concerns about consumer acceptance of beer brewed by genetically modified yeasts (Ishii and Araki, 2016, 2017). Conversely, chemical mutagenesis using methyl benzimidazole 2 yl carbamate (MBC) (Wood, 1982) is, considered as non-GMO method to induce CCNV in *S. pastorianus* and therefore applicable to develop new brewing yeasts.

**FIGURE 8:**
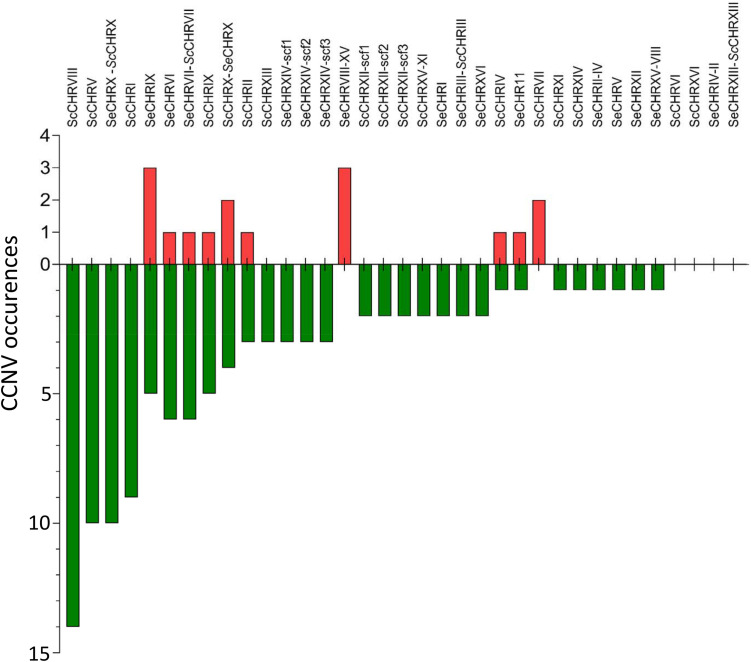
Bar plot of chromosome copy number changes observed in strains generated in this study. Chromosomes were not lost equally in the sequenced strains, untargeted chromosome alterations have been summed over all the analyzed strains for each chromosome and shown in order of observed copy number changes. Instances of loss are shown in green and gain in red.

Accurate measurement of chromosome copy numbers after centromere silencing and MBC treatment generated a unique data set to explore CCNV distribution across mutants quantitatively. Interestingly, loss of chromosomes was more frequent than gain in strains induced for chromosome loss (Figures 3, 8). Theoretically, random chromosome missegregation should cause chromosome loss in the daughter cell and gain in the mother cell or vice versa, leading to equal rates of loss and gain. A higher loss frequency could indicate that chromosome gain is more detrimental than loss, leading to strong selection for cells that have randomly lost rather than gained a chromosome (Santaguida and Amon, 2015b). This is however, contrasting with observation in diploid *S. cerevisiae*, in which chromosome gain (2N to 3N) was more frequent than chromosome loss (2N to 1N), possibly because gain would cause a lower relative change in copy number and thus a lower gene expression impact (Zhu et al., 2014). Chromosome missegregation might also be associated with increased DNA damage and consequently increase chromosome loss after incorporation in a micronucleus as observed in mammalian cells (Santaguida and Amon, 2015a).

Our analysis also revealed that CCNV frequency was different for each chromosome. The loss of *Sc*CHRVIII was significantly more frequent than that of any other (^Fischer exact test^*p*_value_ = 4.2E-2) (Figure 8). Chromosome stability has also been shown to be dependent on the centromere sequence, as the rate of plasmids loss carrying a CEN14 was lower than for plasmids carrying CEN3 which indicates that the frequency of chromosome loss might be associated also the nature of the centromeric region and not only dependent on the genes present on this chromosome. While chromosome stability has also been linked to chromosome size (Kumaran et al., 2013), our data did not corroborate this observation.

The two mutagenesis approaches successfully yielded mutants with improved ethanol tolerance phenotypes. While deep next generation sequencing allows a precise deciphering of the chromosomal copy number, the link between this information and the phenotype remains difficult to establish (Figures 2–4). Mechanisms involving ploidy changes as diploidization or aneuploidy have already been implicated in adaptation of *S. cerevisiae* to high ethanol concentration (Voordeckers et al., 2015; Krogerus et al., 2018; Morard et al., 2019). In a broader context these genetic alterations are more frequently reported (Yona et al., 2012; Bracher et al., 2017; Brickwedde et al., 2017; Harari et al., 2018a, b; Mangado et al., 2018), and changes in full, partial or segmental ploidy, have been shown to contribute effectively to reformatting transcriptome and therefore provide a fast and flexible adaptation to less optimal conditions.

Our study did not completely disentangle the exact contribution of the intrinsic genome instability of the *S. pastorianus* strain and of the mutagenic treatments to obtaining ethanol tolerant variants. Deeper insight could have been gained by submitting the untreated parental strain to the selection procedure experienced by the mutagenized populations. However, based on previous adaptive laboratory evolution strategies to improve ethanol tolerance, generation of tolerant mutants required several hundreds of generations (Voordeckers et al., 2015; Lopandic et al., 2016; Krogerus et al., 2018), contrasting with the reduced number (ca. 25) of generations applied in our study. Further, quantification of CCNV caused by mutagenic treatments showed a significant higher chromosome loss/gain following mutagenesis that would advocate for a key role of the centromere silencing method and MCB treatment in creating a pool of genetic diversity necessary to accelerate the evolution process and the generation of potentially useful variants.

Next to their potential for strain improvement, these methods enabled generation of chromosomal haplotypes by reduction of genomic complexity to chromosome monosomy. The loss of one of the two copies of *Sc*CHRXIII in strain IMS0687 and IMS0703 revealed SNV relative to the parental reference genome that were not acquired de novo but instead resulted from the loss of one of the chromosome copy identifying heterozygous position but also enabling their physical linkage. This illustrates how consensus genome sequence can hide information. In CBS 1483, these two copies were not identical; the lost copy was the one captured in the consensus assembly while the second chromosome variant was revealed by LOH (Figure 7). While heterozygosity at a specific position can be derived from sequencing coverage, allocation of adjacent variants to either chromosome copy (phasing) remains challenging. Sequencing of multiple variants obtained after MBC treatment might be complementary to regular genome sequencing program of polyploid and aneuploid strains to unravel chromosome haplotypes.

In conclusion, despite intrinsic genome instability, exacerbation of this trait is a suitable approach to generate extensive genetic diversity that when coupled to effective selection and screening represents a potent method for strain improvement.

## Data Availability Statement

The datasets generated for this study can be found in the NCBI (https://www.ncbi.nlm.nih.gov/) under the bioproject accession numbers: PRJNA522669 and PRJNA612191. Variant calling files were made publicly available at the 4TU Centre for data research (https://data.4tu.nl/) under the Digital Object Identifier (doi): 10.4121/uuid:e5bc2cfe-d726-44a1-bc0a-d3a06653694.

## Author Contributions

AG, EK, RR, and AM performed the molecular biology work. AG, SO’H, and PV performed the mutagenesis and selection experiments. EK, SO’H, and PV performed the growth characterization. PT performed inhouse next generation sequencing. AG, EK, and MB performed the bioinformatics analysis. AG, JP, and J-MD conceptualized and supervised the study. NB provided critical feedback throughout the study. AG, EK, and J-MD wrote the manuscript. All authors read and approved the final manuscript.

## Conflict of Interest

The authors declare that the research was conducted in the absence of any commercial or financial relationships that could be construed as a potential conflict of interest.

## Footnotes

1 http://www.westerdijkinstitute.nl/

2 https://www.ncbi.nlm.nih.gov/

3 https://data.4tu.nl/
