# Supplementary material for: Improving Industrially Relevant Phenotypic Traits by Engineering Chromosome Copy Number in Saccharomyces pastorianus
Source: Front Genet. 2020 Jun 3;11:518. doi: 10.3389/fgene.2020.00518 (PMC7283523; doi:10.3389/fgene.2020.00518)
Supplement: Supplementary file 1 [file Table_1.DOCX]

Table S1: Primers used in the study

| Primers | Sequence 5’to 3’ | description |
| --- | --- | --- |
| 9857 | GAGATCTGTTTAGCTTGCCTCGTCCCCGCCGGGTCACCCGGATTAGTCGAAGTCGGCCGTG | Forward primer CHR8CER-L |
| 9858 | GACTGTCAAGGAGGGTATTCTGGGCCTCCATGTCGCTGGCCACCACCTCTCTACTATTATCTAGATGC | Reverse primer CHR8CER-L |
| 9859 | TACCTCTATACTTTAACGTCAAGGAGAAAAAACCATCAGAATACACCCTCAGGTTGCTATGAC | Forward primer CHR8CER-R |
| 9860 | GGAGACCGGCAGATCCGCGGCCGCATAGGCCACTAGTGGAGATTTAGGGGCTTCTTCACAG | Reverse primer CHR8CER-R |
| 10088 | GAGATCTGTTTAGCTTGCCTCGTCCCCGCCGGGTCACCCGAAACAAGTTGCGAGACGGTG | Forward primer CHR10CER-L 2 |
| 10089 | GACTGTCAAGGAGGGTATTCTGGGCCTCCATGTCGCTGGCCCGAAGAATTAGTGAATCAATCTCAC | Reverse primer CHR10CER-L 2 |
| 9863 | TACCTCTATACTTTAACGTCAAGGAGAAAAAACCATCAGACCTGGAAGGCTTACCTTATCTATGC | Forward primer CHR10CER-R |
| 9864 | GGAGACCGGCAGATCCGCGGCCGCATAGGCCACTAGTGGAGTGAATAGCCATGCTGGCTG | Reverse primer CHR10CER-R |
| 9865 | GAGATCTGTTTAGCTTGCCTCGTCCCCGCCGGGTCACCCGTGGGAAGCCAAAACCTTACG | Forward primer CHR12CER-L |
| 9866 | GACTGTCAAGGAGGGTATTCTGGGCCTCCATGTCGCTGGCAAGGAAGTAGATGATGGAAAATAAGG TC | Reverse primer CHR12CER-L |
| 9867 | TACCTCTATACTTTAACGTCAAGGAGAAAAAACCATCAGATCCTTTTCTTTTAGCGTCTTGCC | Forward primer CHR12CER-R |
| 9868 | GGAGACCGGCAGATCCGCGGCCGCATAGGCCACTAGTGGACTTCGTGGAAGATTGCCATC | Reverse primer CHR12CER-R |
| 9869 | GAGATCTGTTTAGCTTGCCTCGTCCCCGCCGGGTCACCCGTGAAGATTCGTTAGATCTGCTGG | Forward primer CHR14CER-L |
| 9870 | GACTGTCAAGGAGGGTATTCTGGGCCTCCATGTCGCTGGCCAGCCCACCACAATTCGATT | Reverse primer CHR14CER-L |
| 9871 | TACCTCTATACTTTAACGTCAAGGAGAAAAAACCATCAGAGCGCGCTGATTTAGCAGTAAAATC | Forward primer CHR14CER-R |
| 9872 | GGAGACCGGCAGATCCGCGGCCGCATAGGCCACTAGTGGACTGCTCCACGGGCTACTTAC | Reverse primer CHR14CER-R |
| 9873 | GAGATCTGTTTAGCTTGCCTCGTCCCCGCCGGGTCACCCGAGAGCCATTTGCTGTAGGGG | Forward primer CHR3EUB-L |
| 9874 | GACTGTCAAGGAGGGTATTCTGGGCCTCCATGTCGCTGGCTACAACCGGACCTTATGTCGG | Reverse primer CHR3EUB-L |
| 9875 | TACCTCTATACTTTAACGTCAAGGAGAAAAAACCATCAGACCACTCTCGTAAACATACTTTAATCTC | Forward primer CHR3EUB-R |
| 9876 | GGAGACCGGCAGATCCGCGGCCGCATAGGCCACTAGTGGAAGATCCTGAATATTCTACCGTACTGC | Reverse primer CHR3EUB-R |
| 9877 | GAGATCTGTTTAGCTTGCCTCGTCCCCGCCGGGTCACCCGAGATTAGTCGAAGTCGGCCG | Forward primer CHR8EUB-L |
| 9878 | GACTGTCAAGGAGGGTATTCTGGGCCTCCATGTCGCTGGCTCGAGTTCCTGAAGCTTCTACG | Reverse primer CHR8EUB-L |
| 9879 | TACCTCTATACTTTAACGTCAAGGAGAAAAAACCATCAGAACTAGTTTACCTTACATGCTCCAGACATT ATG | Forward primer CHR8EUB-R |
| 9880 | GGAGACCGGCAGATCCGCGGCCGCATAGGCCACTAGTGGATTCCGTTGGAATGGGCTTCC | Reverse primer CHR8EUB-R |
| 9881 | GAGATCTGTTTAGCTTGCCTCGTCCCCGCCGGGTCACCCGAACGTTTCTGAAGAAAAGAAAGG | Forward primer CHR10EUB-L |
| 9882 | GACTGTCAAGGAGGGTATTCTGGGCCTCCATGTCGCTGGCGCAATTATGTGCGGTGTCTACC | Reverse primer CHR10EUB-L |
| 9889 | GAGATCTGTTTAGCTTGCCTCGTCCCCGCCGGGTCACCCGAGGGGAGGCTGGGTTAATATC | Forward primer CHR14EUB-L |
| 9890 | GACTGTCAAGGAGGGTATTCTGGGCCTCCATGTCGCTGGCAGAGATTCCAGAACATGTGGTC | Reverse primer CHR14EUB-L |
| 9891 | TACCTCTATACTTTAACGTCAAGGAGAAAAAACCATCAGATCCTCAAGTACCCACTGCCC | Forward primer CHR14EUB-R |
| 9892 | GGAGACCGGCAGATCCGCGGCCGCATAGGCCACTAGTGGACGCCCGTGT | Reverse primer CHR14EUB-R |
| 11044 | GAGATCTGTTTAGCTTGCCTCGTCCCCGCCGGGTCACCCGGGTCTTATACGCCAGTCAGCC | Forward primer CHR15-8 EUB-L |
| 11045 | GACTGTCAAGGAGGGTATTCTGGGCCTCCATGTCGCTGGCCGTATCAAATACATACGCTATGGCC | Reverse primer CHR15-8 EUB-L |
| 11046 | TACCTCTATACTTTAACGTCAAGGAGAAAAAACCATCAGATCTTGATTAAAAGCTGTCTGATCTTCGTG | Forward primer CHR15-8 EUB-R |
| 11047 | GGAGACCGGCAGATCCGCGGCCGCATAGGCCACTAGTGGACTAAATTCTTTGACCGCCCATAGG | Reverse primer CHR15-8 EUB-R |
| 8439 | GCCAGCGACATGGAGGCCCAGAATAC | Forward primer amdSPgal |
| 8440 | TCTGATGGTTTTTTCTCCTTGACGTTAAAGTATAG | Reverse primer amdSPgal |
| 8441 | TCCACTAGTGGCCTATGCGGCC | Forward primer pUG Backbone |
| 8442 | CGGGTGACCCGGCGGGGAC | Reverse primer pUG Backbone |
| 10090 | TACCTCTATACTTTAACGTCAAGGAGAAAAAACCATCAGACACCCTTGAAAGAGCCAACTG | Forward primer CHR10EUB-R 2 |
| 10091 | GGAGACCGGCAGATCCGCGGCCGCATAGGCCACTAGTGGAGTCTCTTCTTCCCAAATGTATTTGA | Reverse primer CHR10EUB-R 2 |
| 9885 | GAGATCTGTTTAGCTTGCCTCGTCCCCGCCGGGTCACCCGCCAGATATCCCAAATTAAAGGG | Forward primer CHR12EUB-L |
| 9886 | GACTGTCAAGGAGGGTATTCTGGGCCTCCATGTCGCTGGCAGACTTCTCCTTAATGGAGTTGGG | Reverse primer CHR12EUB-L |
| 9887 | TACCTCTATACTTTAACGTCAAGGAGAAAAAACCATCAGATGTAAGAATGTGACATGAGAATGATC | Forward primer CHR12EUB-R |
| 9888 | GGAGACCGGCAGATCCGCGGCCGCATAGGCCACTAGTGGAGGAAACGCTATACAAAGGCCC | Reverse primer CHR12EUB-R |
| 8624 | ATCACCTAATTAAGGGTTCTC | pUGamdS amplifier FW2 |
| 8623 | GGTCGCTATACTGGAGCTCTCGAGAACCCTTAATTAGGTGATACGGATTAGAAGCCG | FW Pgal fragment V2 |
| 8436 | CGGCAGATCCGCGGCCGCATAGGCCACTAGTGGATCTGATGGTTTTTTCTCCTTGACG | Reverse primer Pgal fragment |
| 8443 | GAGATCTGTTTAGCTTGCCTCGTCCCCGCCGGGTCACCCGAATATGAAGAACTTATAGAGCTATCTAG | Left homology arm scaffold 20 forward |
| 8444 | GACTGTCAAGGAGGGTATTCTGGGCCTCCATGTCGCTGGCAAGGTTATTGAATGCGG | Left homology arm scaffold 20 reverse |
| 8557 | TACCTCTATACTTTAACGTCAAGGAGAAAAAACCATCAGAATGAATATTGCCAATGACTG | Right arm scaffold 20 FW 2 |
| 8510 | GGAGACCGGCAGATCCGCGGCCGCATAGGCCACTAGTGGATAACATTCCAGGATTTAATAGG | Right arm scaffold 20 RV 2 |
| 8451 | GAGATCTGTTTAGCTTGCCTCGTCCCCGCCGGGTCACCCGAGGAATACACACCTTTACCC | Left homology arm scaffold 29 forward |
| 8452 | GACTGTCAAGGAGGGTATTCTGGGCCTCCATGTCGCTGGCACATATTATTTAGCCTTTTTATCCT | Left homology arm scaffold 29 reverse |
| 8453 | TACCTCTATACTTTAACGTCAAGGAGAAAAAACCATCAGAAAACTATAAAAAAAACTTTGCC | Right homology arm scaffold 29 forward |
| 8454 | GGAGACCGGCAGATCCGCGGCCGCATAGGCCACTAGTGGAAGCCTTTTCTAACTTACTTTCA | Right homology arm scaffold 29 reverse |
| 9893 | GTTGTGGAAATGTAAAGAGCCCC | PGAL check genome FW |
